# Circadian Rhythm Shapes the Gut Microbiota Affecting Host Radiosensitivity

**DOI:** 10.3390/ijms17111786

**Published:** 2016-10-26

**Authors:** Ming Cui, Huiwen Xiao, Dan Luo, Xin Zhang, Shuyi Zhao, Qisheng Zheng, Yuan Li, Yu Zhao, Jiali Dong, Hang Li, Haichao Wang, Saijun Fan

**Affiliations:** 1 Tianjin Key Laboratory of Radiation Medicine and Molecular Nuclear Medicine, Institute of Radiation Medicine, Chinese Academy of Medical Sciences and Peking Union Medical College, 238 Baidi Road, Tianjin 300192, China; huiwenxiaokeke@163.com (H.X.); luoxiaobaipumc@163.com (D.L.); zhangxinxin201600@163.com (X.Z.); sukizhao1221@163.com (S.Z.); qishengzheng@126.com (Q.Z.); liyuan_115@126.com (Y.L.); zhaoyupumc@163.com (Y.Z.); dongjiali66@126.com (J.D.); lh880712@sina.com (H.L.); 2 Department of Emergency Medicine, North Shore University Hospital; Laboratory of Emergency Medicine, the Feinstein Institute for Medical Research, 350 Community Drive, Manhasset, NY 11030, USA; hwang@nshs.edu

**Keywords:** circadian rhythm, intestinal bacterial composition, irradiation

## Abstract

Modern lifestyles, such as shift work, nocturnal social activities, and jet lag, disturb the circadian rhythm. The interaction between mammals and the co-evolved intestinal microbiota modulates host physiopathological processes. Radiotherapy is a cornerstone of modern management of malignancies; however, it was previously unknown whether circadian rhythm disorder impairs prognosis after radiotherapy. To investigate the effect of circadian rhythm on radiotherapy, C57BL/6 mice were housed in different dark/light cycles, and their intestinal bacterial compositions were compared using high throughput sequencing. The survival rate, body weight, and food intake of mice in diverse cohorts were measured following irradiation exposure. Finally, the enteric bacterial composition of irradiated mice that experienced different dark/light cycles was assessed using 16S RNA sequencing. Intriguingly, mice housed in aberrant light cycles harbored a reduction of observed intestinal bacterial species and shifts of gut bacterial composition compared with those of the mice kept under 12 h dark/12 h light cycles, resulting in a decrease of host radioresistance. Moreover, the alteration of enteric bacterial composition of mice in different groups was dissimilar. Our findings provide novel insights into the effects of biological clocks on the gut bacterial composition, and underpin that the circadian rhythm influences the prognosis of patients after radiotherapy in a preclinical setting.

## 1. Introduction

The Earth’s rotation on its axis leads to sophisticated day–night cycles, which shape the physiology of almost all living organisms [1]. The mounting list of physiological processes governed by the circadian clock covers metabolism, hormone secretion and body temperature, feeding behavior, and cardiac function, all of which exhibit daily oscillations [2,3]. Modern human lifestyles, including activities such as shift work, nocturnal social activities, and jet lag, increase the risk of developing metabolic syndromes, cardiovascular diseases, and cancer, owing to the disorder of the circadian clock [4]. Recent epidemiological and clinical investigations report that more than 50% of all patients with cancer receive radiotherapy at some point in the course of their disease [5]; meanwhile, radiation exposure in a mass casualty setting is a serious military and public health concern [6]. However, it was previously unknown whether the circadian clock affects the radiosensitivity of mammals.

With an estimated composition of 100 trillion cells, the human body is home to microorganisms, which are typically dominated by bacteria [7], especially in the gastrointestinal (GI) tract, where microbes are at their greatest abundance. The multifarious interactions between the host and microbes define health and disease [8]. Inter-individual variations in the microbiome impact multiple human pathologies, from metabolic disorders to cancer. For instance, preclinical studies describe that cardiovascular disease and metabolic syndrome are accompanied by changes in the composition of gut microbiota [9,10,11]. In this regard, exploring the factors that underlie shifts in the composition and function of gut microbiota are important in order to understand disease processes and to identify new targets for treatment. Accordingly, studies focused on these events have experienced a renaissance, and demonstrate that age, genetics, environment, circadian rhythm, and diet have implications in the alteration of gut microbiota composition [12,13]. However, whether the circadian rhythm-shaped intestinal bacterial community influences the radiosensitivity of mammals remains poorly understood.

In this study, we aim to untangle the effects of circadian rhythm on enteric bacterial composition structure and radiosensitivity using a mouse model. Intriguingly, our data demonstrated that disturbance of the circadian rhythm decreased the number of observed species of gut bacteria in the mouse model. More importantly, the mice that experienced aberrant day/night cycles were more sensitive toward gamma-ray exposure. Thus, our findings provide new insights into the effects of biologic clocks on changes in the composition of gut bacteria, and note the importance of circadian rhythm in patients undergoing radiotherapy.

## 2. Results

### 2.1. Circadian Rhythm Disturbance Shapes Intestinal Bacterial Composition in a Mouse Model

**Figure 1:**
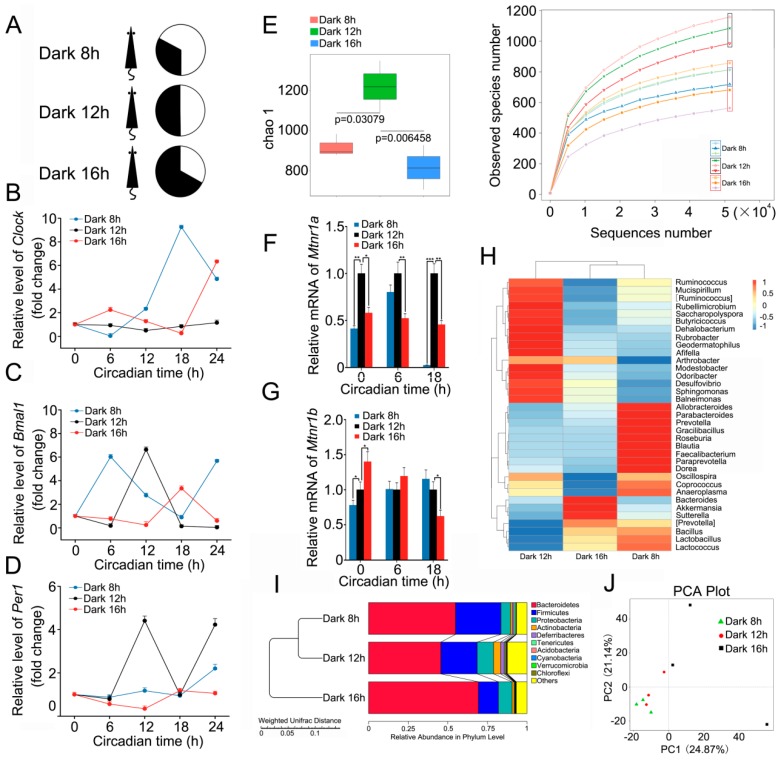
Circadian rhythm disorder alters the intestinal flora composition in a mouse model. (**A**) The diagram shows the experimental dark/light cycles of mice; (**B**–**D**) The expression of *Clock* (**B**), *Bmal1* (**C**), and *Per1* (**D**) was examined by qRT-PCR, using peripheral blood of mice from different cohorts; (**E**) The observed species number of intestinal bacteria in mice from different cohorts were assessed using 16S RNA sequencing, *n* = 3 per group; (**F**,**G**) The expression of *Mtnr1a* (**F**) and *Mtnr1b* (**G**) was examined by qRT-PCR, using peripheral blood of mice from different cohorts; *** *p* < 0.005; ** *p* < 0.01; * *p* < 0.05, Student’s *t*-test. (**H**) The difference in enteric bacterial structure at the genus level, among mice, forming different groups, was assessed using 16S high throughput sequencing, *n* = 3 per group. The color bar denotes the z-scores; (**I**) The relative abundance of bacteria at the phylum level was measured among mice from the three groups using 16S high throughput sequencing, *n* = 3 per group; (**J**) Principal component analysis (PCA) was performed on mice from the three groups using 16S high throughput sequencing, *n* = 3 per group. Statistically significant differences are indicated: Wilcoxon rank sum test.

C57BL/6 mice were separated into three cohorts, and then individually housed in 8 h dark/16 h light, 12 h dark/12 h light, and 16 h dark/8 h light cycles (Figure 1A). After four weeks, quantitative real-time polymerase chain reaction assay validated that the rhythmic expression of circadian locomotor output cycles kaput (*Clock*), aryl hydrocarbon receptor nuclear translocator like (*Bmal1*), and period circadian clock 1 (*Per1*) in peripheral blood (PB) was different in the different groups (Figure 1B–D), thereby indicating that a shift in day-night cycles alters the circadian rhythm of animals. Then, 16S RNA sequencing revealed that, compared with the 12 h dark/12 h light cohort, there were much fewer observed species of enteric bacteria in the other two groups (Figure 1E). Given that gut microbiota is sensitive to melatonin [14], we assesses the expression of *Mtnr1a* and *Mtnr1b*, the receptors of melatonin, at different circadian times. Intriguingly, the expression of *Mtnr1a* and *Mtnr1b* was different among the three cohorts (Figure 1F,G), suggesting that the alteration of intestinal microbiota might depend on circadian rhythm-shaped expression of *Mtnr1a* and *Mtnr1b*. In addition, the relative abundance in the genus level (or phylum level) of *Lactobacillus* (or Bacteroidetes) was lower in 12 h dark/12 h light cohort than in both the other cohorts (Figure 1H,I) At the phylum level, mice housed in 12 h dark/12 h light cycle harbored higher relative abundance of Deferribacteres (Figure 1I). Principal component analysis (PCA) showed that the gut bacterial composition profile substantially varied in mice housed in different day-night cycles (Figure 1J). Together, our observations demonstrate that day–night cycles overtly affect the intestinal bacterial community.

### 2.2. Circadian Rhythm Disorder Impairs the Radioresistance of Mice

**Figure 2:**
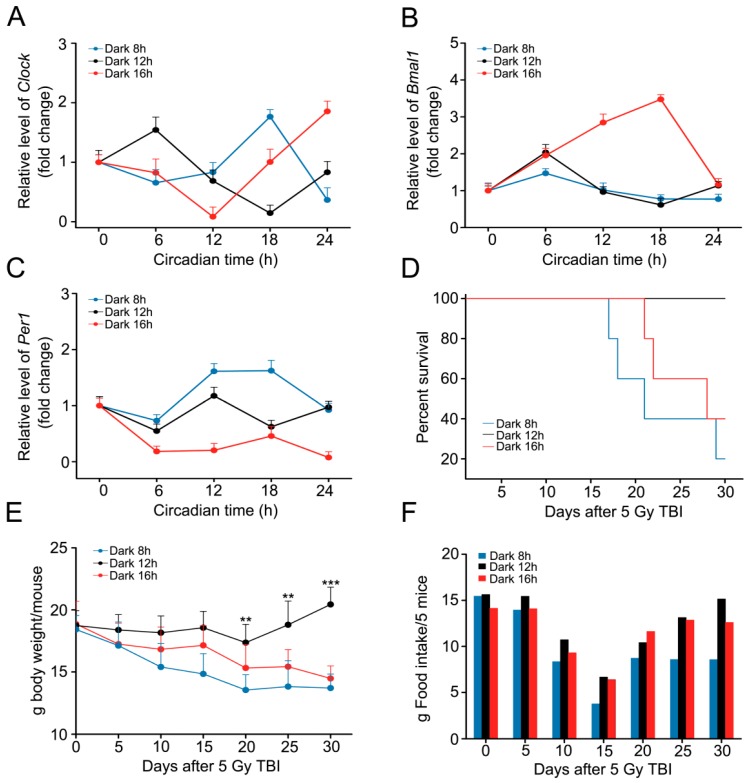
Circadian rhythm disorder impairs the radioresistance of mice. (**A**–**C**) The expression level of *Clock* (**A**), *Bmal1* (**B**), and *Per1* (**C**) was examined by qRT-PCR using the peripheral blood of mice from different cohorts after irradiation; (**D**) Kaplan-Meier survival analyses of mice from the three cohorts, after irradiation, were performed. *p* < 0.05 using a log-rank test between the 12 h dark/12 h light group and the 8 h dark/16 h light (or 16 h dark/8 h light) group, *n* = 10 per group; (**E**) The body weight of mice from the three groups, after irradiation, were measured every 5 days, *n* = 10 per group; (**F**) The food intake of five mice was assessed every five days. Statistically significant differences are indicated: *** *p* < 0.005; ** *p* < 0.01; Student’s *t*-test. TBI, total body irradiation.

To investigate the effects of circadian rhythm perturbation on radiosensitivity, we preformed total body irradiation exposure (5 Gy) using a mouse model. Then, the mice were housed in the three aforementioned day-night cycles. The rhythmical expression of *Clock*, *Bmal1*, and *Per1* was altered in PB from mice housed in 16 h dark/8 h light and 8 h dark/16 h light cycles (Figure 2A–C). More importantly, the survival rates of mice from 16 h dark/8 h light and 8 h dark/16 h light cycle cohorts (about 40% and 20%, respectively) were significant lower than those of mice from the 12 h dark/12 h light cycle group (100%) (Figure 2D). The survival rate of irradiated mice from 12 h dark/12 h light cycle group was decreased after three months, however, the survival rate of irradiated mice from the other two cohorts was 0%. Meanwhile, the body weight of mice from 12 h dark/12 h light cycle cohort was increased slightly; however, they declined dramatically in the other two groups (Figure 2E), indicating that circadian rhythm disorder impairs the radioresistance of mice. Food intake was assessed in the three groups, and mice from the 8 h dark/16 h light cycle cohort ate the least feed (Figure 2F).

### 2.3. Circadian Rhythm Affects the Structure of Gut Bacterial Composition after Irradiation

**Figure 3:**
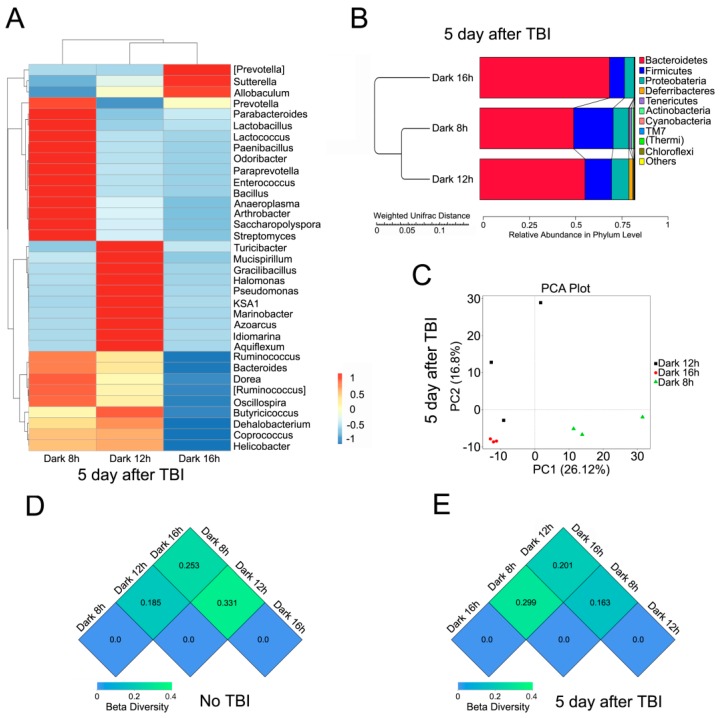
Circadian rhythm affects the structure of gut bacterial composition after irradiation. (**A**) The differences in intestinal bacterial structure at the genus level among irradiated mice from the three cohorts was validated using 16S high throughput sequencing, *n* = 3 per group. The color bar denotes the z-scores; (**B**) The relative abundance of bacteria at the phylum level was examined among irradiated mice from the three groups using 16S high throughput sequencing, *n* = 3 per group; (**C**) Principal component analysis was performed on irradiated mice from the three groups using 16S high throughput sequencing; (**D**,**E**) The β diversity among mice, without (**D**) or with (**E**) irradiation, was analyzed using 16S high throughput sequencing, *n* = 3 per group.

Next, we examined the gut bacterial community of mice in the three groups, after total body irradiation (TBI), using high throughput sequencing. The intestinal microbiota profiles were extremely different in the three cohorts. In detail, the relative abundance of *Lactobacillus*, at the genus level, was much higher in the 8 h dark/16 h light cycle bred mice, compared with the other two groups (Figure 3A) at the genus level, whereas Bacteroidetes were adverse at the genus and phylum levels (Figure 3A,B). PCA revealed that mice housed in different day-night cycles harbored diverse gut bacterial compositions after TBI exposure (Figure 3C). β diversity analysis revealed that the gut bacterial community was more similar in mice in 8 h dark/16 h light and 12 h dark/12 h light cycles, with or without irradiation (Figure 3D,E).

### 2.4. Circadian Rhythm Governs the Response of Gut Bacterial Composition Shift to Irradiation

**Figure 4:**
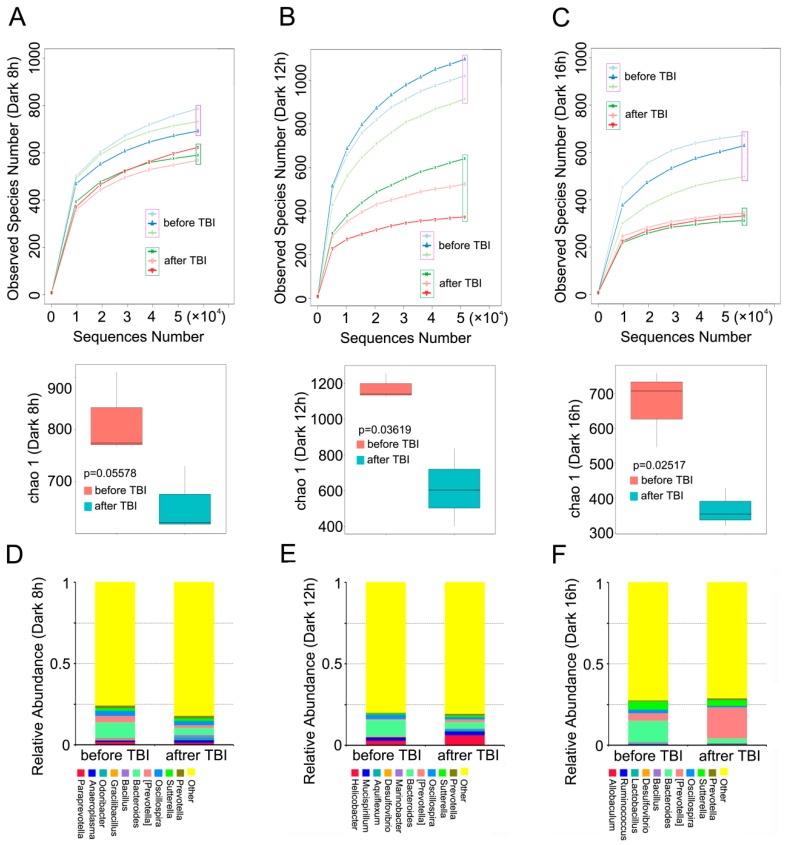
Circadian rhythm governs the response of gut bacterial composition shift to irradiation. (**A**–**C**) The number of observed species of enteric bacteria in mice housed in 8 h dark/16 h light (**A**), 12 h dark/12 h light (**B**), and 16 h dark/8 h light (**C**) cycles was assessed using 16S RNA sequencing, *n* = 3 per group; (**D**–**F**) The relative abundance of the top 10 bacteria at the phylum level in mice housed in 8 h dark/16 h light (**D**), 12 h dark/12 h light (**E**), and 16 h dark/8 h light (**F**) cycles was measured using 16S RNA sequencing, *n* = 3 per group. Statistically significant differences are indicated: Wilcoxon rank sum test.

To better understand the mechanism by which circadian rhythm affects the radiosensitivity of mice, we analyzed the alterations in gut bacterial community of mice housed in the aforementioned day-night cycles, before and after irradiation. 16S RNA sequencing analysis showed that irradiation exposure decreased the number of observed species in all three cohorts (Figure 4A–C). At the genus level, however, the relative abundance of Bacteroidetes declined after irradiation in the three groups (Figure 4D–F), whereas irradiation induced high levels of relative abundance of (Prevotella) in mice housed in 12 h dark/12 h light and 16 h dark/8 h light cycles, and the reverse results were obtained in the other cohort (Figure 4D–F), thereby indicating that day-night cycles, mediated by irradiation, shape the alterations in gut bacterial composition.

## 3. Discussion

Biological clocks have evolved as an adaptation to life on a rhythmic planet, synchronizing physiological processes to the environmental day–night cycle. Given that light is a central modulator of circadian rhythm [15,16], we bred mice under three different dark-light cycles to change their intrinsic biological clocks. Biological rhythms are tunable by environmental stimuli, and all eukaryotic cells potentially exhibit sophisticated cellular circadian oscillations [17]. Thus, we assessed the circadian rhythms of mice housed in 8 h dark/16 h light, 12 h dark/12 h light, and 16 h dark/8 h light cycles using peripheral blood. As expected, the light-dark cycles impaired the expression of rhythmical genes, indicating that environmental stimuli might disturb the circadian rhythm of animals at a molecular level. Behavioral fluctuations and intrinsic time keeping mechanisms have been reported to govern daily endocrine oscillations [18]. Mounting studies underpin the strong links between circadian rhythms and the immune system, suggesting that circadian rhythms are employed as crucial regulators of specific immune functions [19]. Clinically, circadian dysfunction might exacerbate neurodegenerative disorder processes [20], the development of cancer [21], and drive the onset of cardiovascular diseases [22]. Disruption of sleep is an established risk factor for diabetes and is well known to promote systemic metabolic dysfunction [23]. Inspired by these studies, we evaluated the gut bacterial community of mice in the three aforementioned cohorts. Intriguingly, circadian rhythm disorder decreased the observed number of species and also shaped the gut flora profile, indicating that circadian rhythm is a key modulator in maintaining intestinal microflora balance. To further uncover the underlying mechanism by which circadian rhythm shapes the gut microbiota, we examined the expression levels of *Mtnr1a* and *Mtnr1b* of mice from the three groups at different circadian times. As expected, the expression of *Mtnr1a* and *Mtnr1b* were different, suggesting that *Mtnr1a* and *Mtnr1b* might be involved in the circadian rhythm-shaped gut bacterial community.

The gastrointestinal tract is home to microorganisms that live in a symbiotic relationship with the host, and are key determinants of health and disease by influencing nutrient absorption, barrier function, and immune development [24,25]. Recent investigations revealed that changes in gut microbiota and their metabolites have implications in many inflammatory and gastrointestinal diseases, such as asthma, arthritis, functional gastrointestinal disorders, inflammatory bowel disease, and wound-healing [26]. Epidemiological evidence bolsters that flora disequilibrium is associated with the development of cancer, particularly gastric and colorectal cancers [27], indicating that gut bacteria profoundly affect the physiopathologic processes of the host. Radiotherapy is a cornerstone of modern management of malignancies, and more than half of all patients with cancer receive this treatment at some point during the course of their disease [5]. Accordingly, we examined the effect of circadian rhythm on the radioresistance using a mouse model. Our observations showed that disturbance of biological clocks, not only shaped gut bacterial composition, but also altered the radiosensitivity of the hosts. Notably, there are no significant differences in food intake between mice housed in 12 h dark/12 h light and 16 h dark/8 h light cycles, indicating that nutrient supplementation is not determinant for circadian-rhythm-impaired radiosensitivity. The irradiation-altered intestinal bacterial communities were dependent on the light/night cycles experienced by the hosts, suggesting that a shift of gut microbiota might play pivotal roles in circadian-rhythm-impaired radiosensitivity. Together, our findings underpin that circadian rhythm intertwines with prognosis after radiotherapy in an enteric-bacteria-dependent fashion. The underlying mechanism required further study.

In summary, we identified that biological clock disorder induced intestinal flora disequilibrium, resulting in impairment of the radioresistance of hosts. Disturbance of the circadian rhythm dramatically reduced the number of observed bacterial species. More importantly, circadian-rhythm-disorder-shaped gut bacterial composition impaired host radioresistance in a nutrient-supply-independent fashion. Thus, our findings provide new insights into circadian-rhythm-disorder-mediated physiopathologic processes, and shed light on the importance of biological clocks in prognosis after radiotherapy.

## 4. Materials and Methods

### 4.1. Animals

Six- to eight-week-old male C57BL/6J mice were purchased from Vital River (Beijing, China); mice were housed in the Specific Pathogen Free level animal facility at the Institute of Radiation Medicine (IRM), the Chinese Academy of Medical Sciences (CAMS). Male C57BL/6 mice were housed in individual cages in a temperature-controlled room (ambient temperature 22 ± 2 °C air humidity 40%–70%), and had free access to food and water, according to NIH guidelines for the use and care of live animals, and were approved by our Institutional Animal Care and Use Committee (IACUC) (Permit Number 1526, 7 April 2015). All procedures and animals handlings were performed following the ethical guidelines for animal studies. The mice were randomly separated into three groups, and were individually housed in 16 h dark/8 h light, 12 h dark/12 h light, and 8 h dark/16 h light cycles. All animals used in this study were male mice from a pure C57BL/6 genetic background.

### 4.2. Irradiation Study

A Gammacell^®^ 40 Exactor (Atomic Energy of Canada Lim, Chalk River, ON, Canada) was used in all experiments. Nonanesthestized mice were immobilized in a specific steel chamber, and radiation dose was monitored by a dose rate meter. In this study, male (about 20 g in weight) mice were treated with total abdominal irradiation (TAI) with a single dose of 5 Gy γ-ray, at a rate of 1.0 Gy/min. After irradiation, the mice were returned to the animal facility for daily observation and treatment as described below. Animal body weight was assessed every five days.

### 4.3. Quantitative Real-Time Polymerase Chain Reaction (qRT-PCR)

Total RNA was separated from peripheral blood from mouse vena ophthalmica every six hours using Trizol (Invitrogen, Carlsbad, CA, USA), according to manufacturer protocols. cDNA was produced by using poly(A)-tailed total RNA and reverse transcription primer with ImPro-II Reverse Transcriptase (Promega, Madison, WI, USA), according to the manufacturer’s instructions. qRT-PCR was performed according to the instructions of the Fast Start Universal SYBR Green Master (Rox) (Roche Diagnostics GmbH, Mannheim, Germany). *GAPDH* was used as control. The primers used in this study were:
*Clock*: forward, AGTTAGGGCTGAAAGACGGC, reverse, TAGAGGAGGCAGAAGGAGTTGG;*Bmal1*: forward, AGATAAACTCACCGTGCTAAG, reverse, AGATAAACTCACCGTGCTAAG;*Per1*: forward, AGGGTGAGCCTTGTGCCAT, reverse, AGATGGTGTAGTAGAGCCATAG;*Mtnr1a*: forward, TGGCTGTTTACCCTTATCCC, reverse, AAACCACCACTGCTATCGTG;*Mtnr1b*: forward, GTGTCATTGGCTCTGTCTTCA, reverse, TGTGCTGGCTGTCTGGATGAA;*GAPDH*: forward, TGTTTCCTCGTCCCGTAGA, reverse, CAATCTCCACTTTGCCACTG.

### 4.4. Bacterial Diversity Analysis

Fresh stool samples were collected at about 10:00 a.m. and were stored at −80 °C before use. DNA was extracted from the stool using a Power Fecal^®^ DNA Isolation Kit (MoBio, Carlsbad, CA, USA). The DNA was recovered with 30 mL of buffer included in the kit. The 16S ribosomal RNA (rRNA) gene was analyzed to evaluate bacterial diversity using Illumina Hiseq (Novogene Bioinformatics Technology Co., Ltd., Beijing, China). The primers used in this study were 515F, GTGCCAGCMGCCGCGGTAA, and 806R, GGACTACHVGGGTWTCTAAT.

### 4.5. Statistical Analyses

The data are presented as the means ± SEM with respect to the number of samples (*n*) in each group. Statistical significance between multiple treatment groups was determined using analysis of variance (ANOVA), Wilcoxon rank sum test, and Tukey’s *t*-test. Survival rates were analyzed using the Kaplan-Meier survival test. Results with *p* < 0.05 were considered to be statistically significant.

## 5. Conclusions

Modern lifestyle, such as shift work, nocturnal social activities and jet lag, disturbs the circadian rhythm and has implication for diverse diseases. Here, we identify that biologic clock disorder induces intestinal flora disequilibrium and impairs the radioresistance of hosts in a nutrient supply-independent fashion. Thus, our findings provide new insights into circadian rhythm disorder-mediated physiopathologic process, and shed a light on the importance of biologic clocks on the prognosis after radiotherapy.
